# Assessment of private health sector prescribing patterns and adherence to prescription format using World Health Organization core drug use indicators in Addis Ababa, Ethiopia

**DOI:** 10.1186/s40545-022-00408-0

**Published:** 2022-03-01

**Authors:** Getahun Asmamaw, Nahu Ejigu, Dinksew Tewihubo, Wondim Ayenew

**Affiliations:** 1 Department of Pharmacy, Arba Minch University, Arba Minch, Ethiopia; 2 Pharmacist, Arabisa Health Center, Addis Ababa, Ethiopia; 3 Department of Pharmacy, Medda Wolabu University, Bale Robe, Ethiopia; 4 Department of Pharmaceutics, University of Gondar, Gondar, Ethiopia

**Keywords:** Ethiopia, Prescribing patterns, WHO core drug use indicators, Private healthcare sector

## Abstract

**Background:**

Currently, the private healthcare sector's role in healthcare delivery is growing in Ethiopia. However, there are limited studies on private healthcare sector drug use patterns. This study aimed to evaluate the private healthcare sector prescribing practices and adherence to prescription format, using some of the World Health Organization (WHO) core drug use indicators in Addis Ababa, Ethiopia.

**Methods:**

A retrospective cross-sectional study design was used to collect quantitative data from prescriptions prescribed and dispensed by private healthcare sectors in the Lemi-Kura sub-city, Addis Ababa. The study was conducted from June to July 2021. The WHO criteria were used to evaluate prescribing and prescription completeness indicators. Prescriptions, kept for the last 1 year that were prescribed between January 1, 2020, to January 1, 2021, by private drug outlets, were analyzed. Simple random and systematic sampling procedures were employed in selecting drug outlets and prescriptions, respectively.

**Results:**

Of a total of 1,200 prescriptions, 2,192 drugs were prescribed and the average number of drugs per prescription was 1.83. Generic names, antibiotics, injections, and drugs on the Ethiopian essential medicines list accounted for 77.4, 63.8, 11.5, and 80.6% of all prescriptions, respectively. Among the patient identifiers, the patient card number (54.3%), weight (2.3%), and diagnoses (31.7%) were less likely to be completed. In terms of the drug-related information, the dosage form (35.5%) was the least likely to be completed. Only 36.6 and 25.8% of prescriptions contained the names and qualifications of the prescribers, respectively. It was difficult to obtain prescription papers with the dispenser identifier.

**Conclusion:**

The study findings indicated prescribing and prescription completeness indicators all considerably deviated from WHO standards and hence unsuitable. This situation could be critical since a similar pattern is reported from public healthcare sectors, which might imply the extent of non-adherence to WHO core drug use standards. Consequently, it could play a considerable role in increasing irrational medicine use in Ethiopia.

**Supplementary Information:**

The online version contains supplementary material available at 10.1186/s40545-022-00408-0.

## Background

Medicines play a crucial role in the delivery of quality healthcare services. The appropriate use of medications is critical to the improvement of a patient's disease condition [1]. The improper use of medicine, on the other hand, may have unintended consequences for the patient. For example, side effects, unwelcome costs, sickness mistreatment, and antibiotic resistance are some drawbacks of irrational medicine use [2, 3]. The World Health Organization (WHO) defines rational medicine use as patients' use of medications for the specified clinical ailment, in the proper dose, in the appropriate dosage forms, for an adequate amount of time at a minimal cost [4].

In Ethiopia, irrational prescribing and poor compliance with prescription format practices are reported, particularly from public healthcare sectors [5–8]. However, it is suggested that similar outcomes are uncommon in the country's private healthcare sector prescribing and dispensing practice [7]. On the other hand, the private healthcare sector's involvement in healthcare delivery is expanding in Ethiopia [7, 9]. As a reason, a high number of prescriptions are projected to come from the private sector [9]. Globally, an irrational prescription is common in the private sector. For example, only a small percentage of drugs are prescribed by their nonproprietary names, and inaccuracy is designated as a violation of WHO's key drug use indicators [1, 10, 11].

Therefore, a periodic examination of medication prescribing and dispensing practices in a private healthcare facility could aid in identifying specific medication use issues, educating practitioners on rational medicine prescription and dispensing, and providing policymakers with important data for revising medicine related policies. This study aimed to evaluate the private healthcare sector's prescribing practices and adherence to fundamental prescription information, using some of the WHO core drug use indicators in Addis Ababa, Ethiopia. The findings of this study will aid in prioritizing the most important intervention areas for rational medicine use. It will also help as an initial point for researchers who want to do more research into the factors that influence drug usage patterns in the private healthcare sector.

## Materials and methods

### Study area and period

Addis Ababa, the capital of Ethiopia has 11 administrative zones. In the city, nearly 94% of healthcare facilities are private [7]. The city has more than 25 and 759 private for-profit hospitals and clinics, respectively. Similarly, more than 308 pharmacies, 249 drug stores, and one rural drug vendor are hosted in the city [12]. The sub-city of Lemi-Kura is east of the Addis Ababa municipal administration. It is a newly reorganized administrative zone composed of ten woredas, which has been active since 2020 [13]. The research was carried out at eight private community pharmacies and drug stores in the Lemi-Kura sub-city. Only two drug outlets from each of the four woredas (a total of eight) were chosen purposely due to the outbreak of the coronavirus disease (COVID-19) pandemic during the study period. The study was carried out from June to July 2021.

### Study design

A retrospective cross-sectional study design was used to collect quantitative data from prescriptions prescribed from private healthcare institutions. The study was carried out in randomly selected private community pharmacies and drug stores placed at the Lemi-Kura sub-city. The WHO criteria were used to evaluate both types of indicators (prescribing and prescription completeness indicators) [14].

### Sampling

Both simple random sampling and systematic sampling procedures were employed in this investigation [15]. The research site (the Lemi-Kura sub-city administration) was chosen by lottery initially, followed by four woredas in the sub-city. Then, from each of the woredas, one pharmacy and one drug store were selected. The outlets were chosen for each category, beginning with a randomly selected outlet and continuing in a 5-outlet interval. A total of eight drug outlets, four pharmacies (named Gammel, l6, Werramo, and Dudu), and four drug stores (named Yodahe, Anbessa, Hasset, and Belay) were chosen. Following that, private healthcare prescriptions were filtered out by excluding prescriptions from public healthcare facilities, prescriptions that were illegible or unclear, prescriptions that only contained non-drug products (such as medical supplies, fluids, or nutritional products), and prescriptions that were issued before or after the specified prescription time. Prescriptions, kept for the last 1-year that were prescribed between January 1, 2020, to January 1, 2021 in private drug outlets were selected.

A systematic random sampling technique was then utilized to choose prescriptions obtained from private healthcare facilities by taking every five prescriptions at drug outlets. The sampling interval was obtained by dividing 6,000 (total number of prescriptions fulfilled the criteria) by 1,200 (desired sample size). The sample size was determined based on the WHO guidelines, at least 600 encounters should be included in the sample [10]. As a result, prescribing indicators were evaluated retrospectively using 1,200 prescriptions from eight different pharmacies and drug stores (150 prescriptions from each).

### Data collection

In this study, WHO-designed data collecting forms based on criteria were used. The data on prescribing and completeness indicators were collected by three well-trained pharmacists by observing prescriptions. Data from the prescription paper were filled out on a well-structured questionnaire by the collectors.

### Data analysis and interpretation

The data were entered and analyzed using SPSS V 20.0 statistical software. In the statistical analysis, the indicators were reported as frequencies, averages/means, percentages, and proportions. The findings were interpreted using WHO completeness and prescribing indicator standards.

### Completeness and prescribing indicators

The WHO criteria for completeness and prescribing indicators were used in this study. As a result, common factors such as the prescriber's identity and drug-related information were used to assess the prescription completeness. As a result, the adherence proportion of patient information (age and gender, weight, and card number), treatment information (drug name, drug strength, dose, frequency, duration, dosage form, diagnosis, and how to use), and prescriber and dispenser information (full name, qualification, date of prescription/dispensing, signature of the prescriber) in the total included prescription was calculated. Prescribing indicator statistics also included the average number of drugs prescribed for each issue (used as a degree of polypharmacy), the percentage of prescriptions written by generic name, antibiotic prescriptions, injectable prescriptions, and prescriptions from the Ethiopian Essential Medicine List (EML) [16]. Thus,

1. The degree of polypharmacy = $\frac{\mathrm{Total no}.\mathrm{ of different drug products prescribed}}{\mathrm{Total no}.\mathrm{ of prescriptions polled}}$
2. ─ Drug combinations prescribed for a single health issue were counted as one.
3. Drugs prescribed by generic name (%) = $\frac{\mathrm{No}.\mathrm{ of drugs prescribed by generic name }}{\mathrm{Total no}.\mathrm{ of drugs prescribed}}\mathrm{ X }100.$
4. Antibiotic prescriptions (%) = $$\frac{\mathrm{No}.\mathrm{ of antibiotic prescribed }}{\mathrm{Total no}.\mathrm{ of prescriptions polled}}\mathrm{ X }100.$$
5. Injection prescriptions (%) = $$\frac{\mathrm{No}.\mathrm{ of injections prescribed }}{\mathrm{Total no}.\mathrm{ of prescriptions polled}}\mathrm{ X }100.$$
6. Drugs prescribed from EDL (%) = $\frac{\mathrm{No}.\mathrm{ of drugs prescribed from EDL }}{\mathrm{Total no}.\mathrm{ of drugs prescribed }}\mathrm{X }100.$

The completeness indicators that were measured included the proportion of prescriptions that contained:

1. Patient information (such as age and sex, address, full name) was calculated by dividing the number of completed encounters in which such information was prescribed by the total number of encounters surveyed, multiplied by 100.
2. Treatment information (such as dosage form, strength, label, total amount) was calculated by dividing the number of completed encounters in which such information was prescribed by the total number of encounters surveyed, multiplied by 100.
3. Professionals’ information (such as name, address, telephone number of the prescriber, signature, or qualification of the prescriber) was calculated by dividing the number of completed encounters in which such information was prescribed by the total number of encounters surveyed, multiplied by 100.

### Operational definitions

#### Private healthcare sectors

The healthcare delivery firms owned by individuals or limited liability companies that are primarily limited to "for-profit" purposes.

#### Drug outlets

Are dispensaries that have been legally registered as pharmacies or drug stores.

#### Combination of drugs

Triple therapy for *Helicobacter pylori*-induced peptic ulcer, for example, is regarded as one.

#### Ethiopian essential medicines list

The collection of essential drugs, or those that meet the population's most pressing healthcare needs. In this study, it was used interchangeably with another term "Ethiopian essential drug list" [14].

#### Antibiotics

Pharmaceuticals like penicillin and other groups of antibacterial, anti-infective drugs (such as dermatological and ophthalmological agents), as well as antidiarrheal drugs such as streptomycin, neomycin, and metronidazole, in the context of this study [5, 17].

#### Generic drug

Globally recognized International Nonproprietary Name (INN) that is stated as a public property. The 2014 Ethiopian EML was used to determine if drugs were generic or brand names [16].

#### Adherence to the basic prescription format

Completing prescription format of some basic information such as patient, treatment, and information of prescriber and dispenser [18].

## Results

### WHO core prescribing indicators

In the sampled prescriptions (1200), a total of 2192 medicines were prescribed. An average of 1.83 medications were prescribed per encounter (Table 1). Out of all prescriptions, 690 (57.5%) contained two or more drugs per prescription, ranging from one to six medicines per encounter. Generic drugs made up 1431 (65.3%) of all prescribed medications. At least one antibiotic was prescribed in 766 (63.8%) of all prescriptions. Injections were prescribed in 137 (11.5%) of the prescriptions examined. The majority of the drugs (*n* = 1730; 78.9%) were prescribed from the Ethiopian EML.

**Table 1:** WHO prescribing indicator of prescription practice (N = 1200 prescriptions)

Indicators	Total drugs/prescriptions	Average/ percent	Standard values [10]
Average number of drugs per prescription		1.83	1.6–1.8
Drugs prescribed by generic name	1431	65.3%	100%
Prescriptions with an antibiotic prescribed	766	63.8%	20–26.8%
Prescriptions with injection prescribed	137	11.5%	13–24.1%
Number of prescribed drugs from Ethiopian EML	1730	78.9%	100%

Based on source of prescriptions, most of (793; 66%) the collected prescriptions were prescribed from clinics. An average of 1.78 and 1.85 medication per patient were prescribed in hospitals and clinics, respectively (Table 2). However, an average of 0.81 and 0.72 antibiotic per encounter were issued in hospitals and clinics, respectively.

**Table 2:** The prescription practice based on the sources of prescription in private healthcare facilities (N = 1,200 prescriptions)

Prescribing indicators	Source of prescription			
	Hospital		Clinic	
	Total drugs/prescriptions	Average/percent	Total drugs/prescriptions	Average/percent
Average number of drugs per encounter		1.78		1.85
Drugs prescribed by generic name	425	19.4%	1006	46%
Average number of antibiotics per encounter	0.81	0.699	0.72	0.667
Prescriptions with injection prescribed	28	2.3%	109	9.1%
Number of prescribed drugs from Ethiopian EML	607	27.7%	1123	51%

#### Completeness of the prescription

In this study, the completeness of the prescription was evaluated, including patient information, medication information, and professional information (Table 3). Patient-related information such as age, sex, and the full name of the patient was completed on more than 95% of the sample prescriptions. On the other hand, the patients' weight, was the least likely to be filled on prescriptions. More than half of the prescriptions did not include information on the medication such as the dosage form, diagnosis, or how to take medicines.

**Table 3:** Completeness of prescriptions among prescriber and dispenser private healthcare facilities (N = 1,200 prescriptions)

Patient information		Treatment information		Professional information			
				Prescribers		Dispensers	
Variable	*N* (%)	Variable	*N* (%)	Variable	*N* (%)	Variable	*N* (%)
Full name	1187 (98.9)	Drug name	1193 (99.4)	Full name	439 (36.6)	Full name	109 (9.1)
		Drug strength	1059 (88.3)				
Sex	1156 (96.3)	Dose	1022 (85.2)	Qualification	309 (25.8)	Qualification	15 (1.3)
Age	1143 (95.3)	Frequency	1170 (97.5)	Date of prescription	957 (79.8)	Date of dispensing	8 (0.7)
Weight	28 (2.3)	Duration	1084 (90.3)	Signature	1,127 (94)	Signature	322 (26.8)
Card No	651 (54.3)	Dosage form	426 (35.5)	All completed per prescription*	270 (22.5)	All completed per prescription*	0 (0.0)
All completed per prescription*	16 (1.3)	Diagnosis	380 (31.7)				
		How to use	1048 (87.3)				
		All completed per prescription*	100 (8.4%)				

*Fully completed patient, treatment or professional related information forms per prescription

On the professional related information form, the date of prescription 957 (79.8%) and signature 1127 (94%) were more likely to be filled out by the prescribers. However, only 270 (22.5%) of prescriptions had all of the required information on the prescriber form. Interestingly, no dispenser was found to have filled out all of the required dispenser-related information per prescription.

## Discussion

In this study, the WHO core drug use indicators and prescription completeness were used to assess the practice of private healthcare sectors in Addis Ababa in terms of drug use patterns and prescription completeness. This study looked at 2192 medicines that were prescribed on 1,200 prescriptions. It was shown that the average number of drugs per issue and the percentage of drugs prescribed by generic name, antibiotic, injection, and drugs from Ethiopian EML were 1.83, 65.3, 63.8, 11.5, and 78.9%, respectively. Moreover, only 1.3, 8.4, 22.6% of prescriptions were completed with all of the basic patient, treatment and professional-related information, respectively.

As per the findings of this study, the average number of medications ordered per prescription from the private healthcare sector in Addis Ababa was 1.83. This is slightly higher than the WHO standard comparison (1.6–1.8) [10]. This result is consistent with two prior studies, Ayenew et al. and Bahiru et al. that conducted systematic reviews at the country level in Ethiopia. They found that the pooled number of drugs per prescription exceeded WHO standards in the public healthcare facilities, accounting for 1.96 and 2.14, respectively [19, 20]. Moreover, a polypharmacy was also recorded in Addis Ababa and other parts of the country. For example, in Tikur Anbesa Specialized Hospital (TASH) in Addis Ababa, the average number of medicines per prescription was found to be 1.89 [21]. Similarly, according to the studies from other parts of the country, drug per prescription amounts 1.9, 2.34, 2.2, and 2.13 in southern, eastern, northern, and southwest regions of Ethiopia, respectively [5, 22–24]. The higher number of drugs per encounter were also reported across public and private healthcare facilities in Asian and sub-Saharan African (SSA) countries such as Vietnam (3.85), India (3.7), Pakistan (4.1), Saudi Arabia (2.36), and South Africa (3.7), Sudan (2.3), Zambia (2.5), Sierra Leone (3.8), Nigeria (3.5) [4, 25–31]. Furthermore, a study in Tanzania indicated private healthcare facilities are prescribing more drugs per encounter than public healthcare facilities [32]. The study concluded that interventions are required to combat the role of private healthcare facilities in irrational medicine use. Although the factors have not yet been investigated enough in SSA countries, a constraint in prescribers having appropriate therapeutic training, prescriber behavior, variation in the healthcare delivery system, differences in socioeconomic profiles, as well as morbidity and mortality characteristics of the population might have contributed [5, 10].

In the present study, the percentage of medications prescribed by generic name in the assessed private healthcare sectors was substantially below (65.3%) the WHO standard of 100% prescribing generic drugs [10]. However, according to various Ethiopian studies, generic drugs make up the majority of Ethiopian prescriptions. For example, according to a review study of Bahiru et al. the pooled percentage of pharmaceuticals administered by generic name was found to be 93.5% (89.13%–97.96%) in Ethiopian public healthcare facilities [20]. Similarly, studies from public healthcare facilities in Addis Ababa (88.5%) [33], Hawassa (98.7%) [5], among selected healthcare facilities in eastern Ethiopia (97%) [23], North-west Ethiopia (88%) [34], and South-west Ethiopia (77.3%) [18], also showed higher percentages of generic prescribing than the current study. The higher score of public sectors could indicate that the public healthcare sector receives medication mainly from the Ethiopian Pharmaceutical Supply Agency (EPSA), which purchases more generic pharmaceuticals [35]. However, according to studies conducted in Ethiopia's southwest region, participants stated that brand prescribing has certainly grown [5, 18]. Similar studies in Vietnam, India, Pakistan, Saudi Arabia, South Africa, and Sudan also reported that private healthcare sectors prescribed more brand names than public ones [4, 25–28]. Accordingly, the current study's lower rate of generic prescriptions from private healthcare sectors could be attributed to a range of circumstances in our context. For example, there is no mandatory policy requiring private healthcare providers to purchase from public suppliers, which could lead to prescribers easily influenced by factors such as the prescriber's characteristics, the cost of the medicine, and the marketing and promotion of importers and pharmaceutical companies [36].

The percentage of prescriptions containing antibiotics was 63.8% in this study, approximately three times the WHO limit (20–26.8%) [10]. Similarly, Ethiopian public healthcare facilities also show a high prevalence of antibiotic prescription, although it was lower than the findings of this study [5, 19, 20, 24, 37]. In Ethiopia, antibiotics may be prescribed excessively for a variety of reasons. For example, various studies have found that Ethiopia has a high frequency of infectious illnesses [38], a lack of adherence to treatment protocols [20], and a disparity in healthcare professional understanding of rational antibiotic prescription [39]. Antimicrobial resistance is also on the rise, and there have been instances of hospitalization and mortality as a result of it [40]. As a result, it is possible that Ethiopia's antimicrobial resistance danger is being exacerbated by the over-prescription of antibiotics [41]. Antibiotic resistance has a considerable impact on medicine accessibility and healthcare financing in Ethiopia because of the high prevalence of poverty and limited resources [42]. Furthermore, the percentage of antibiotic prescription of the current study was higher than studies from Sudan (63%), Zambia (53.7%), Uganda (53%), Swaziland (54%), and Jordan (61%). [31, 43].

In the current study, the percentage of encounters issued with injections were was 11.5%, which is lower than the WHO criterion (13–24.1%) [10]. However, according to the reports of Ayenew et al. and Bahiru et al., the pooled Ethiopian injection use in public healthcare facilities were within the WHO standard range accounts 18.3% and 13.2%, respectively. [19, 20] Likewise, studies done in Addis Ababa (19.31%) and Mekelle (23.6%) were also reported an acceptable WHO standard of injection prescription [34, 44]. However, other similar studies in TASH (53.1%), Dessie (9.0%), and the two public healthcare districts of Northern Ethiopia (3.6%) reported above and below the WHO standard, respectively [21, 37, 45]. Several reasons could be mentioned for the variability among the healthcare facilities in Ethiopia. Professional and patient-related intervention efforts are among the most regularly mentioned factors of prescribing injections [46]. For example, according to the findings of one study in Ethiopia, patients have a lower preference for injection dosage forms due to concerns about contamination and the belief that other dosage forms are equally effective [47]. The other possible reason could be the relative higher cost of injections than other dosage forms particularly oral medications in Ethiopia [48]. This might made prescribers and patients to prefer for less costly dosage forms [49]. This could indicate the lower prevalence of injections in the private sector might be linked to a commitment to their client's preference. Consequently, according to the study in Nepal, a lower incidence of therapeutic injection prescribing was recommended since it lowers the danger of infection via the parenteral route as well as the cost of treatment. [50] The percentage of injection prescription of the current study are higher than those reported in Nigeria (9%) and Vietnam (34.2%), but lower figure than Sudan (27.09%), Zambia (11.8%), Pakistan (19%), and Nepal (30%) [25, 31, 50–53].

In this study, the majority of drugs (n = 1,730; 78.9%) prescribed in the private healthcare sectors were on the Ethiopian EDL. But, this was lower than the WHO standard of 100% for prescription of medicine on EDL [10]. This score was lower than similar studies done in public healthcare facilities in Ethiopia [5, 19, 20, 24]. Similarly, the review study which included 11 SSA countries, revealed that private healthcare facilities were less likely to prescribe drugs on national EML than public healthcare sectors [54]. The lower adherence in private healthcare sectors of SSA countries might be critical since, compliance with the EDL is one of the key tools for a stable national healthcare delivery system. Thus, it could ensure the availability and affordability of quality medicines thereby promoting the rational use of medicines [17, 55].

In this study, patients' full names, ages, and sexes were mentioned in 99, 95.3, and 96.3% of prescription papers, respectively. However, the card number (54.3%) and weight of the patient (2.3%) were less likely to adhere to in this investigation. Similarly, age and sex were well-presented in the study conducted in governmental facilities of South-Eastern Ethiopia, which also revealed that full name, age, sex, and card number were mentioned in 100, 81.8, 76.3, 39.8% of the encounters, respectively [24]. Moreover, study from TASH in Addis Ababa also revealed full name age, sex, weight and card number were mentioned in 94.5, 25.1, 26, 0.3, and 22.4% of the prescriptions, respectively [21]. This might be explained as the majority of prescribers use the patient's name rather than a card number during prescribing medicine. The other possible explanation might be related to the burden flow of clients, which caused them to lean toward what was easiest to provide the service. The finding of this study concerning completing patient-related information (full name age, sex, weight and card number) on prescription paper was roughly better than study reports from Pakistan and India [52, 56].

In the present study, the treatment-related information such as drug name, strength, dose, frequency, duration, and how to use was completed in a range of 85% to 99% of the evaluated prescriptions. However, prescribers were less likely to complete out dosage forms (35.5%) and diagnoses (31.7%). Similarly, Ethiopian public healthcare facilities also reported a lower percentage of adherence to filling out the medication-related information than the WHO standard of completing 100% of medication-related information on prescriptions [5, 10, 11, 57, 58]. Likewise, in Nepal 88, 40, 71, 90, 60, and 37% of prescriptions contained the dosage form, quantity, dose, frequency, strength, and route of administration, respectively. [59] In Pakistan, the name of drug, dose, dosage form, and how to use was mentioned in 100, 88, 97, 97% of prescriptions, respectively [52]. However, the lower rate of adherence to complete treatment-related information on prescriptions was also reported in South Africa [60]. Medication errors, drug-related adverse events, and therapeutic failure could occur when medications are dispensed with insufficient labels [10]. As a result, practitioners are required to be enforced to label each drug that is given to the patients.

In terms of prescriber-related information, only 36.6 and 25.8% of prescribers wrote their name and qualification on prescriptions, respectively. While a higher percentage of prescriptions were found to be filled with their day of prescriptions (79.8%) and prescriber signatures (94%). Moreover, in this study, only 100 (8.4%) of prescriptions were completed with all of the prescriber-related information. Similarly, a lower adherence rate of completing prescriber identifies was also reported in public healthcare facilities in Ethiopia. For example, according to Admassie et al. 33.4, 96.7, 72.6, and 16.1% of prescriptions have the prescriber's name, signature, date, and qualification, respectively [62]. Other similar study in South West Ethiopia reported only 16.4% and 76.3% of all evaluated prescriptions appeared with the name and signature of prescribers [24], comparing with the investigations in Pakistan and Saudi Arabia which illustrated the name of the prescriber in 82% and 83.3% of all evaluated prescriptions, respectively [52, 61]. The findings of this study could indicate the lower adherence to complete the name of prescriber on prescriptions. The poor adherence to prescriber identifiers in Ethiopia could imply the difference in perceptions of the value of prescribing information. Thus, because of this poor practice, identifying the accountable prescriber for any feedback or explanation could be challenging.

Moreover, this study also revealed obtaining prescription papers with the full name, qualification, date of dispensing, and signature of the dispenser was extremely difficult. Only 9.1, 1.3, 0.7, and 26.8% of dispensed prescriptions contained such information, respectively. Interestingly, no dispenser filled out all of this information in a single prescription. Likewise, finding from TASH also revealed the barely dispenser adherence to fill the dispenser-related information on prescription paper [21]. However, in the pediatric emergency unit of a tertiary hospital in Lagos, Nigeria, for example, the dispensers placed their signature following a refill in 92.1% of prescriptions [57]. In the study, because the type and contents of the prescriptions used by the practitioner differed, the preparation and execution of standard prescriptions in all departments and units of the hospital were critical.

According to the study from Vietnam, the private healthcare system has a greatest role in challenging the promotion of rational medicine use by increasing provider-induced drug demand. The provider-induced drug use can be expressed as high-rate prescription of a greater number of drugs per illness and injections than public healthcare facilities, which aimed to build a public trust. The study also revealed the poor regulatory system, lack of reinforcing activities (such as involving private healthcare sectors in providing health insurance), and patient information gap empowered the private sectors to induce unnecessary demand of medicine use in Vietnam [25]. Thus, the findings of this study could also indicate the potential of private healthcare role in increasing the irrational medicine use in Ethiopia. Therefore, further studies such as “the provider–agent impact of private healthcare providers” are needed to be investigated not only in Ethiopia, but also for other countries (particularly developing countries) that requires to encourage private health sector’s role in provision of rational medicine use.

## Limitation of the study

There are some drawbacks to this study. These limitations, however, do not invalidate the study's findings because all of the WHO-recommended procedures were used. Although this study attempted to cover all of the WHO core drug use and prescription indicators, the smaller sample size, as well as the study's limited scope, may limit the generalizability of these specific components to the general population. Multi-institutionally and sampling approaches could help to overcome the limitation in generalizability. This study used prescribing indicators and prescription completeness to measure the drug utilization pattern and prescription format adherence. Studies based on such a method might not indicate the presence or absence of rational prescribing and good dispensing practices, as those indicators do not necessarily explain the involvement of the prescriber and patient in the process. This study, on the other hand, provided useful information on the private sector's practice of prescribing patterns and adherence to basic prescription information.

## Conclusion

Whereas the average number of drugs per prescription, percentage of drugs prescribed as generics, percentage of prescriptions with antibiotics, and percentage with injections was substantially less than in the Attock study. Whereas the average number of drugs per prescription, percentage of drugs prescribed as generics, percentage of prescriptions with antibiotics, and percentage with injections was substantially less than in the Attock study. Whereas the average number of drugs per prescription, percentage of drugs prescribed as generics, percentage of prescriptions with antibiotics, and percentage with injections was substantially less than in the Attock study. In this investigation, unsatisfactory prescription patterns and adherence to basic prescription information per WHO standards were observed. This situation could be critical since a similar pattern was reported from public healthcare sectors, which might imply the extent of non-adherence to WHO drug use standards which could play a great role in increasing irrational medicine use in the country. Because of the importance of rational medicine use and private sector practice in Ethiopia, health practitioners must be provided with regulatory interventions, continuous monitoring from concerned bodies, and regular training on proper prescribing and dispensing practices to promote rational drug use. Finally, further similar studies in every corner of the country are needed to be done. Moreover, studies which aimed to reveal the factors that determine drug usage patterns in the private healthcare sector and the impact of private health market regarding medication utilization are also needed in our context.

## Supplementary Information

**Additional file 1.** Prescribing core indicators data.

**Additional file 2.** Prescription adherence indicators data.

**Additional file 3.** Prescription data abstraction form.

## Data Availability

In Additional file 1 and Additional file 2, one may find the data that support the result of this article (Additional file 3).

## Abbreviations

EML/EDL: Essential medicines/drugs list
EPSA: Ethiopian pharmaceutical supply agency
FMOH: Federal Ministry of Health of Ethiopia
INN: International nonproprietary name
SD: Standard deviation
SPSS: Statistical package for the social sciences
SSA: Sub-Saharan Africa
TASH: Tikur Anbesa Specialized Hospital
WHO: World Health Organization
