# Supplementary material for: Assessment of private health sector prescribing patterns and adherence to prescription format using World Health Organization core drug use indicators in Addis Ababa, Ethiopia
Source: J Pharm Policy Pract. 2022 Mar 1;15:11. doi: 10.1186/s40545-022-00408-0 (PMC8886342; doi:10.1186/s40545-022-00408-0)
Supplement: Supplementary file 3 — Additional file 3. Prescription data abstraction form. [file 40545_2022_408_MOESM3_ESM.docx]

**Prescription Data Abstraction Form**

**STUDY TITLE: Assessment of private health sector prescribing patterns and adherence to prescription format using World Health Organization core drug use indicators in Addis Ababa, Ethiopia**

Data Collector: ____________________Date Collected: ___________________

Date of Prescription: ________________ Cod Number: ___________________

1. **Eligibility checklist**
2. Was the prescription/ patient encounter written at privet health sectors?

[ ] Yes [ ] No

1. Was the prescription/ patient encounter written between January 1, 2020, to January 1, 2021? [ ] Yes [ ] No
2. Was the prescription/ patient encounter clear to read? [ ] Yes [ ] No
3. Was the prescription/ patient encounter contained at least one drug product? [ ] Yes [ ] No

**NOTE:** All the four Eligibility criteria above MUST be answered ‘YES’ before data abstraction.

**B) Source of prescription**

1. Prescription source [ ] 1=Hospital__________ [ ] 0= clinic ________

**C) Prescribing indicators**

Annex I: Prescribing indicators form for examining the completeness and prescription pattern

| Total number of drugs prescribed | ………………… |
| --- | --- |
| Number of drugs prescribed using generic name | …………………. |
| Any antibiotic prescribed? | 1= Yes 0= No |
| Any injection prescribed? | 1= Yes 0= No |
| Number of drugs prescribed from Ethiopian Essential medicine list (EML) | ………………….. |

**D) Completeness of the prescription**

Annex II: Prescribing indicators form for examining the completeness and prescription pattern

| Patient information | | Treatment information | | Professional information | | | |
| --- | --- | --- | --- | --- | --- | --- | --- |
|  |  |  |  | **Prescribers** | | **Dispensers** | |
| Variable | **N(%)** | **Variable** | **N (%)** | **Variable** | **N(%)** | **Variable** | **N(%)** |
| Full name |  | Drug name, strength, |  | Full name |  | Full name |  |
| Sex |  | Dose |  | Qualification |  | Qualification |  |
| Age |  | Frequency |  | Date |  | Date |  |
| Weight |  | Duration |  | Signature |  | Signature |  |
| Card No. |  | Dosage form |  |  |  |  |  |
|  |  | Diagnosis |  |  |  |  |  |

1. Is the prescription complete considering ALL the 5 criteria? [ 1] = Yes [0] =No
